# Elective implant removal in the upper extremity: only symptomatic patients benefit

**DOI:** 10.1007/s00590-023-03777-7

**Published:** 2023-11-20

**Authors:** Jan Hambrecht, Claudio Canal, Felix Klingebiel, Cyrill Pfammatter, Michel Teuben, Valentin Neuhaus, Hans-Christoph Pape, Yannik Kalbas, Christian Hierholzer

**Affiliations:** 1 Department of Trauma Surgery, University Hospital Zurich, Rämistrasse 100, 8091 Zurich, Switzerland; 2 Department of Surgical Research, Harald Tscherne Laboratory for Orthopaedic and Trauma Research, Zurich University Hospital, Zurich, Switzerland

**Keywords:** Implant removal, Patient satisfaction, Symptoms of discomfort, Clavicle, Olecranon, Distal radius

## Abstract

**Purpose:**

Elective implant removal (IR) in the upper extremity remains controversial. Implants in the olecranon and clavicle are commonly removed for prominence, unlike in the distal radius. Patient-reported symptomatic cannot be verified, and nonspecific discomfort remains unquantified. In this study, indications and outcomes of IR at the clavicle, olecranon and distal radius were evaluated, with a focus on postoperative patient satisfaction.

**Materials and methods:**

In this retrospective, single-center cohort study, patients, who received elective IR of the clavicle, olecranon and distal radius were included. Patients were followed up at least six weeks after IR. Outcomes included patient satisfaction, symptom resolution, and complications.

**Results:**

One hundred and eighty-nine patients were included. Unspecific symptoms of discomfort were the most prevalent indication for IR (48.7%), followed by pain (29.6%) and objective limited range of motion (ROM) (7%). Pain and limited ROM combined was observed in 13.8%. Subjective benefit following IR was described in 54%. Patients with limited ROM (OR 4.7, *p* < 0.001) or pain (OR 4.1, *p* < 0.001) were more likely to experience alleviation of complaints. Patients with unspecific symptoms of discomfort, often did not report improvement. Major complications occurred in 2%. Refractures were detected at the clavicle (3.7%) and at the olecranon (2.5%). Minor complication rate was 5%.

**Conclusion:**

IR is a safe procedure in the upper extremity. Indications based on unspecific symptoms of discomfort have a significant lower rate of patient satisfaction postoperatively. Elective IR should be considered cautiously, if it is driven primarily by unspecific symptoms of discomfort. Patient education is relevant to prevent dissatisfying outcome.

## Introduction

The number of elective IR after surgically treated fractures on the upper extremity is constantly increasing and elective IR accounts for up to 30% of all orthopedic surgeries [1]. Overall, 10–15% of upper extremity injuries receive IR after plate osteosynthesis [2]. The indications for IR are manyfold. While there are several absolute indications for IR, such as nonunion or infection, relative indications include pain, restrictions of movement as well as unspecific and sometimes weather-dependent symptoms of discomfort. Indications for IR in the upper extremity become even more distinct when considering hardware positioned superficially, such as at the olecranon or clavicle, versus hardware surrounded by critical structures like at the distal radius. A study performed by Reith et al. pain and limited ROM were described as the leading reasons for IR [3].

Functional upper extremity motion enables patients to execute activities of daily living; therefore, implant- related functional impairment may result in significant limitations in quality of life. However, the topic of IR is underrepresented in the scientific literature and guidelines as well as evidence-based recommendations are lacking [1]. According to a German study, common postoperative complications of elective IR include wound healing disorders (21%), residual material (12%), and neurovascular injuries (14%) [3]. There is even more controversy about unspecific symptoms of discomfort, which is a common indication for IR. In most cases, unspecific symptoms of discomfort cannot be independently and reliably verified and quantified. The extent and possible adverse effects of persisting hardware can only be determined by taking a thorough patient history. However, since standardized quantifications are missing it remains a therapeutic challenge to derive valid indications for elective IR.

Therefore, we initiated this study to investigate indications, complications, and patient satisfaction after elective IR in consolidated fractures of the upper extremity. We hypothesized, that indications for elective IR based on unspecific symptoms of discomfort may result in similar patient satisfaction, compared to clinically objective indications, such as pain or limited ROM.

## Materials and methods

### Study design

This project is a retrospective cohort study. The reporting in this study adhered to the STROBE (Strengthening the reporting of observational studies in epidemiology) statement [4].

### Ethical considerations

This study was approved by the cantonal ethics committee and was conducted according to the Declaration of Helsinki. (PB_2016-01888). Only patients with written consent were included.

### Study populations

The patients included in this study were operatively treated for elective IR in the upper extremity between 2016 and 2021 at the outpatient clinic of a Swiss Level 1 trauma center. We screened all patients, who presented to our outpatient clinic with radiologically consolidated fractures of the upper extremity, after open reduction and internal fixation with a request for IR. To allow adequate comparability, we decided to limit our cohort to the three most common anatomical regions of IR on the upper extremity (clavicle, distal radius, and olecranon). All patients who received elective IR in different anatomical regions were excluded. In addition, patients were excluded from this study if they were younger than 18 years of age or pregnant, as well as patients without written informed consent or missing data.

### Surgery and aftercare

All patients presented with radiologically consolidated fractures after open reduction and internal fixation of the upper extremity. General anesthesia or plexus anesthesia was used based on patient preference. According to literature, clinical experience and radiological control, criteria and time point for IR were radiographically consolidated fractures and a postoperative interval to the index operation of at least one year for distal radius and olecranon fracture, and two years for clavicle fractures [5]. Anterior- and superior plate osteosynthesis were removed at the clavicle as well as volar plate osteosynthesis at the distal radius. At the olecranon, tension belt- and plate osteosynthesis were removed. All implant removals were performed in an outpatient, “same day surgery” setting. The patients were followed up regularly in our outpatient facility, one day after surgery to perform wound control, and after 6 weeks for a clinical examination and radiological control.

### Clinical evaluation and outcomes

The evaluation of the patients was conducted before and after IR. Indications for IR were evaluated in a clinical examination based on subjective and objective criteria: Objective criteria included clinical restrictions such as triggerable pain or objective limited ROM, while subjective criteria included mainly unspecific symptoms of discomfort.

Our primary outcome assessment was patient satisfaction, which was evaluated at the follow-up visit six weeks after IR. Patient satisfaction was defined as the patient experiencing relief from preoperative complaints. Furthermore, the return to work and the use of pain medication were assessed. Secondary outcomes included complications, and objective improvements based on a clinical examination carried out by a physician of our trauma department. Therefore, we compared the objective preoperative and postoperative ROM for the shoulder, elbow, and wrist using the neutral-zero method. For the shoulder, ab-, adduction, anteversion, retroversion, and external and internal rotation were measured. For the elbow joint, extension and flexion, and for the wrist joint, extension and flexion, pro- and supination, as well as radial- and ulnar- abduction were assessed, as well as full finger extension and fist formation. An improvement in ROM was recorded when postoperative ROM increased by more than 10° compared to the preoperative status.

### Definitions

**Patient satisfaction** was defined as the patient experiencing relief from preoperative complaints. **Pain** was defined as any kind of painkiller required and triggerable complaints. **Limited range of motion** was defined as objective restrictions in the clinical examination. **Unspecific symptoms of discomfort** were defined as patients’ discomfort without objective, triggerable complaints or restrictions and the absence of painkiller use.

### Statistics

Continuous data are presented with mean and standard deviation (SD), and categorical variables are presented with numbers and percentages. Statistical analysis was performed in R (R Core Team (2018). R: A language and environment for statistical computing. R Foundation for Statistical Computing, Vienna, Austria. URL: https://www.R-project.org/).

The ggplot2-package was used for data-visualization. Data were visually tested for normality using histograms. Unpaired student *T* tests were used for parametric data. Nonparametric data were tested using Wilcoxon-Mann–Whitney tests. Binary categorical data were assessed using Fisher’s exact test, and non-binary categorical data using chi-squared test with Yates` correction for continuity. Odds ratios (OR) were calculated using a logistic regression model. The threshold for statistical significance was determined as a *p* value of < 0.05.

## Results

### Study population

**Fig. 1:**
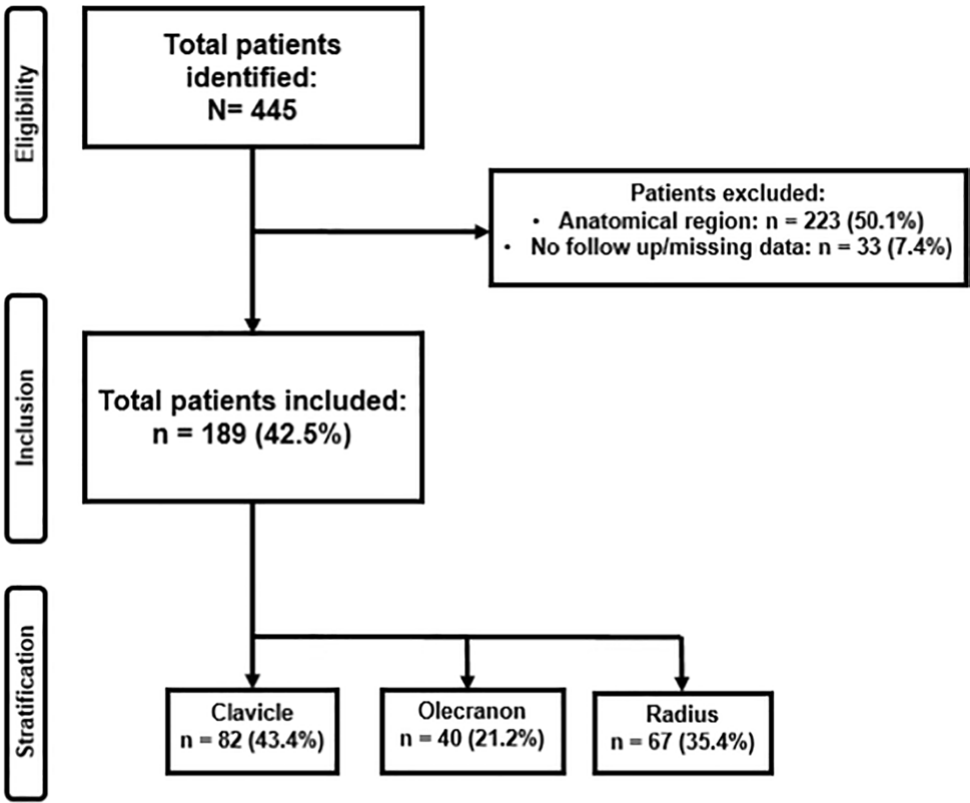
Patient inclusion flowchart

Four hundred and forty-five patients were identified, who received elective IR. Of these, 223 patients (50.1%) were excluded due to anatomical location. 33 patients (7.4%) were excluded because of loss to the follow-up. Overall, 189 patients were included for the final analysis. Of these, 82 patients had an IR of the clavicle, 40 of the olecranon, and 67 of the distal radius. The selection process is visualized in Fig. 1.

### Patient demographics, indications, and procedural parameters

Patient demographics and indications are presented in Table 1. Of the 189 included patients, 76 were female (40.2%) and 113 male (59.7%) with a mean age of 43.7 years (range: 19–77 years) (SD = 15.6). Relevant comorbidities effecting wound healing (e.g., diabetes, kidney failure, peripheral artery disease) were observed in 24.5% of the patients. Smoking was described by 21.2% of all patients.

**Table 1:** Demographics and indications

	Clavicle	Olecranon	Radius
*n*	82	40	67
Demographics			
Age (mean (SD))	42.66 ± (14.89)	48.85 (± 16.12)	41.87 (± 15.69)
Sex = female (%)	17 (20.7)	25 (64.1)	34 (50.7)
Duration of surgery (mean ± SD)	37.90 (± 16.99)	43.05 (± 26.73)	35.32 (± 17.54)
Smoking	17 (20.7)	8 (20.0)	15 (22.4)
Comorbidities	20 (24.4)	13 (33.3)	13 (19.4)
Indication, *n* (%)			
Pain + limited ROM	8 (9.8)	2 (5.0)	5 (7.5)
Limited ROM	6 (7.3)	10 (25.0)	10 (14.9)
Pain	24 (29.3)	12 (30.0)	20 (29.9)
Symptoms of discomfort	44 (53.7)	16 (40.0)	32 (47.8)

**Fig. 2:**
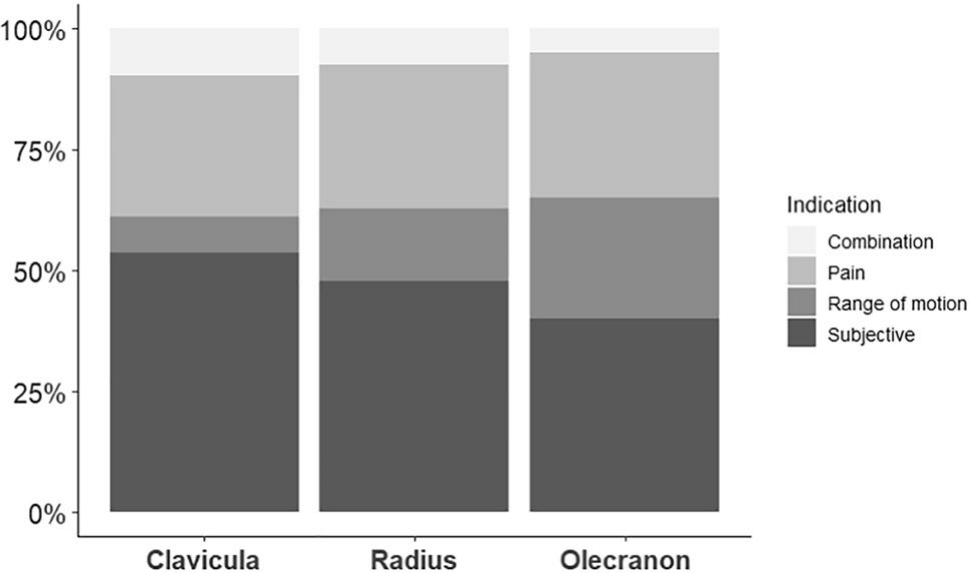
Indications for implant removal stratified by anatomical region

Patients were stratified (clavicle, distal radius, and olecranon) according to their indications for IR, which were: triggerable pain requiring painkillers, objective limited ROM motion or unspecific symptoms of discomfort. This is visualized in Fig. 2.

For IR after clavicle fractures, unspecific symptoms of discomfort (53.7%) were identified as the dominant indication. Pain was reported in 29.3% of patients as the main reason for IR. A painless objectively limited ROM was seen in 7.3% of the included patients. A combination of pain and limited ROM was mentioned in 9.8% of cases.

Regarding IR on the radius, unspecific symptoms of discomfort were observed as the most prevalent reason for IR (47.8%). Pain was described in 29.9% and an objective limited ROM in 14.9% of the patients. A combination of pain and limited ROM was detected in 7.5% of the patients. For olecranon fractures, the main reason for IR was also unspecific symptoms of discomfort (40%). The second-most reason identified for IR was pain (30%). Pain, as well as the combination of pain and an objective limited ROM, was described in 5% of the patients. Sensitivity to cold weather was described only in olecranon (2.5%)—and distal radius fractures (4.5%).

**Fig. 3:**
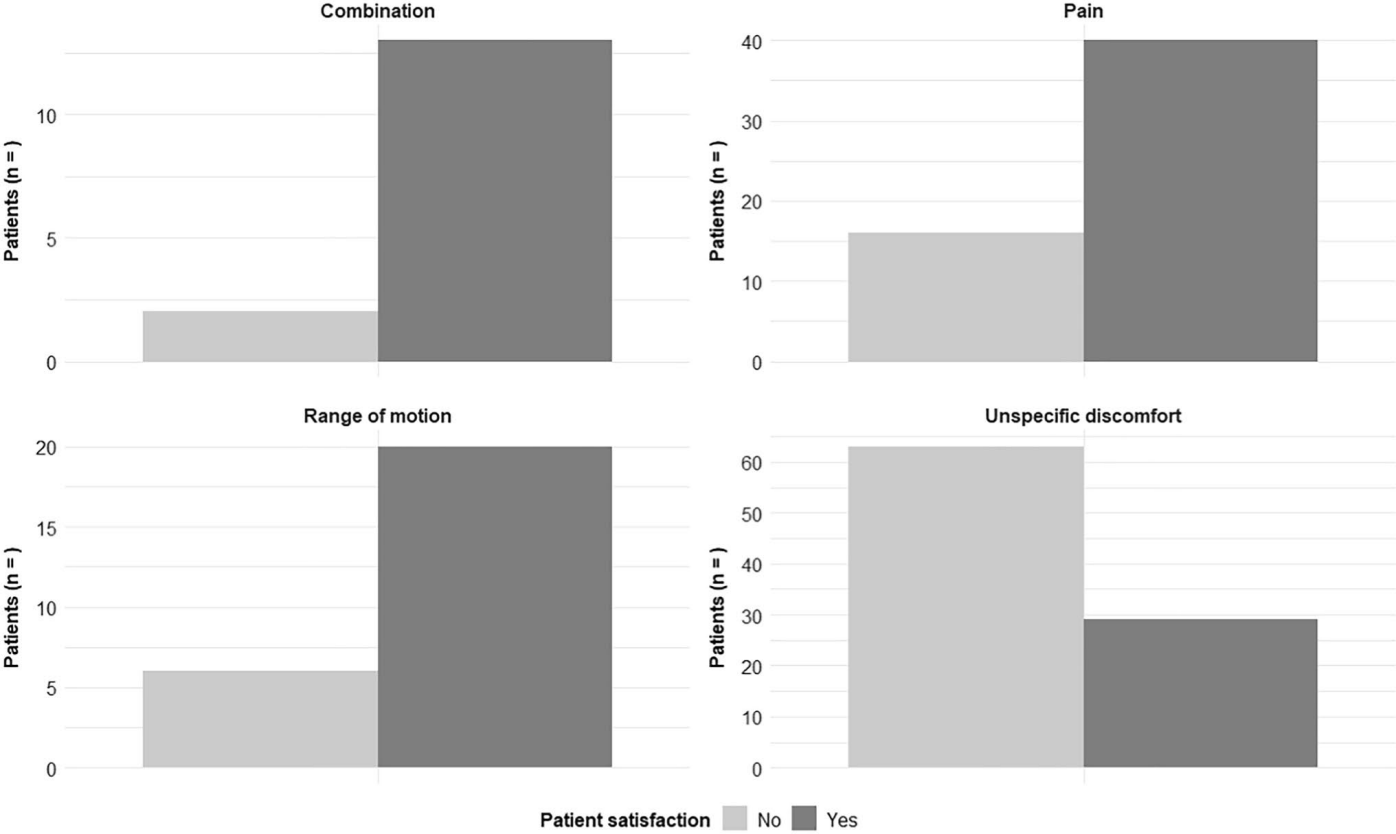
Patient satisfaction after implant removal in view of the indication

**Fig. 4:**
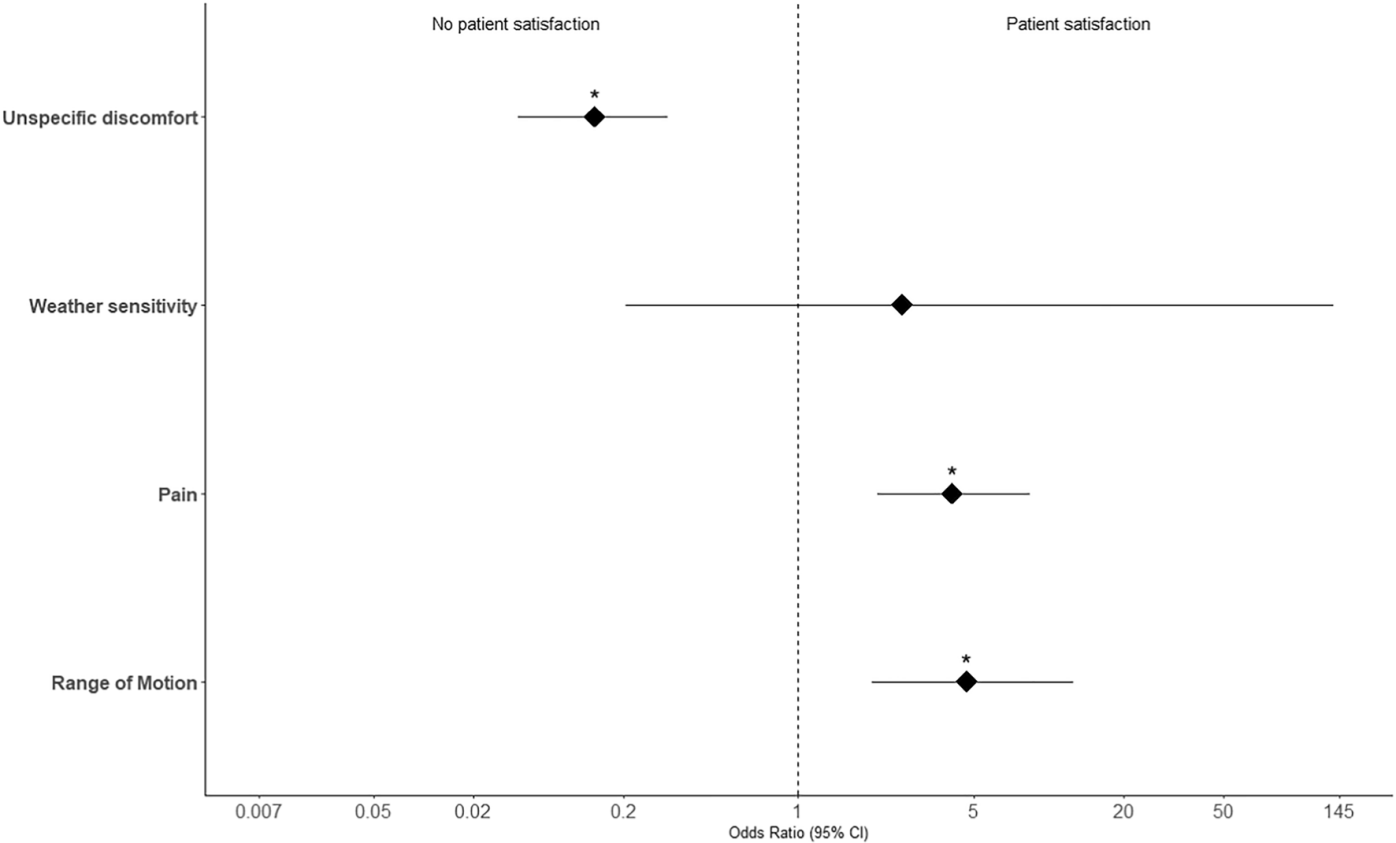
Likelihood of patient satisfaction represented as odds ratio with 95% confidence intervals

Regarding our primary outcome, patient satisfaction was described in 58.8% after IR of the clavicle, 50% after IR of the olecranon, and 49.3% after IR of the radius. We could demonstrate that patient satisfaction following IR correlated with the indication for the procedure (see Table 2 and Fig. 3). Regarding this, most of the increasing patient satisfaction after IR were detected when there was an objective restriction, such as pain (71.4%), a limitation in ROM (76.9%) or a combination of them (86.7%). On the other hand, patients without clinically objective complaints showed lower patient satisfaction after elective IR (30.4%). Odds ratios for a satisfactory outcome were calculated based on the indication (Fig. 4). Patients who showed an objectively limited ROM (OR 4.7, *p* < 0.001) or pain (OR 4.1, *p* < 0.001) were significantly more likely to experience and report subjective alleviation of symptoms after IR of the upper extremity. Patients who reported unspecific symptoms of discomfort prior to IR, were significantly less likely to report postoperative satisfaction (OR 0.15, *p* = 0). A complete overview of the statistical analyses including ORs, Confidence Intervals (CIs) and *p* values is provided in Table 3.

**Table 2:** Intraoperative management and postoperative outcome

	Clavicle	Olecranon	Radius
Intraoperative management, *n* (%)			
Overdrilling	4 (4.9)	2 (5.0)	2 (3.0)
Residual implants	7 (8.5)	4 (10.0)	5 (7.5)
Postoperative outcome, *n* (%)			
Patient satisfaction	**49 (58.8)**	**20 (50.0)**	**33 (49.3)**
Persistent pain	5 (6.1)	7 (17.5)	5 (7.5)
Improvement ROM	16 (19.5)	11 (27.5)	27 (40.3)
Major complications	**3 (3.7)**	**1 (2.5)**	**0 (0)**
Refracture	3 (3.7)	1 (2.5)	0 (0)
Deep infections	0 (0)	0 (0)	0 (0)
Minor complications	**2 (2.4)**	**6 (15.0)**	**2 (3.0)**
Wound healing disorder	2 (2.4)	5 (12.5)	1 (1.5)
Iatrogenic injuries	0 (0)	0 (0)	0 (0)

**Table 3:** Summary of statistical analyses. Odds rations represent the likelihood of patient satisfaction based on indication and complications based on risk- and procedural factors

	Odds ratio	Lower 95% CI	Higher 95% CI	*p*-value
Outcome: Improved patient satisfaction				
Pain	4.114	2.078	8.423	< 0.001
ROM	4.686	1.956	12.555	< 0.001
Subjective complaints	0.153	0.076	0.299	0
Weather sensitivity	2.594	0.204	138.276	0.626
Outcome: Complications				
Smoking	1.541	0.333	5.742	0.499
Comorbidities	0.921	0.156	3.801	1
Overdrilling	1.838	0.038	16.249	0.466
Residual implants	3.366	0.537	15.151	0.102

*CI* confidence interval

### Secondary outcomes

**Fig. 5:**
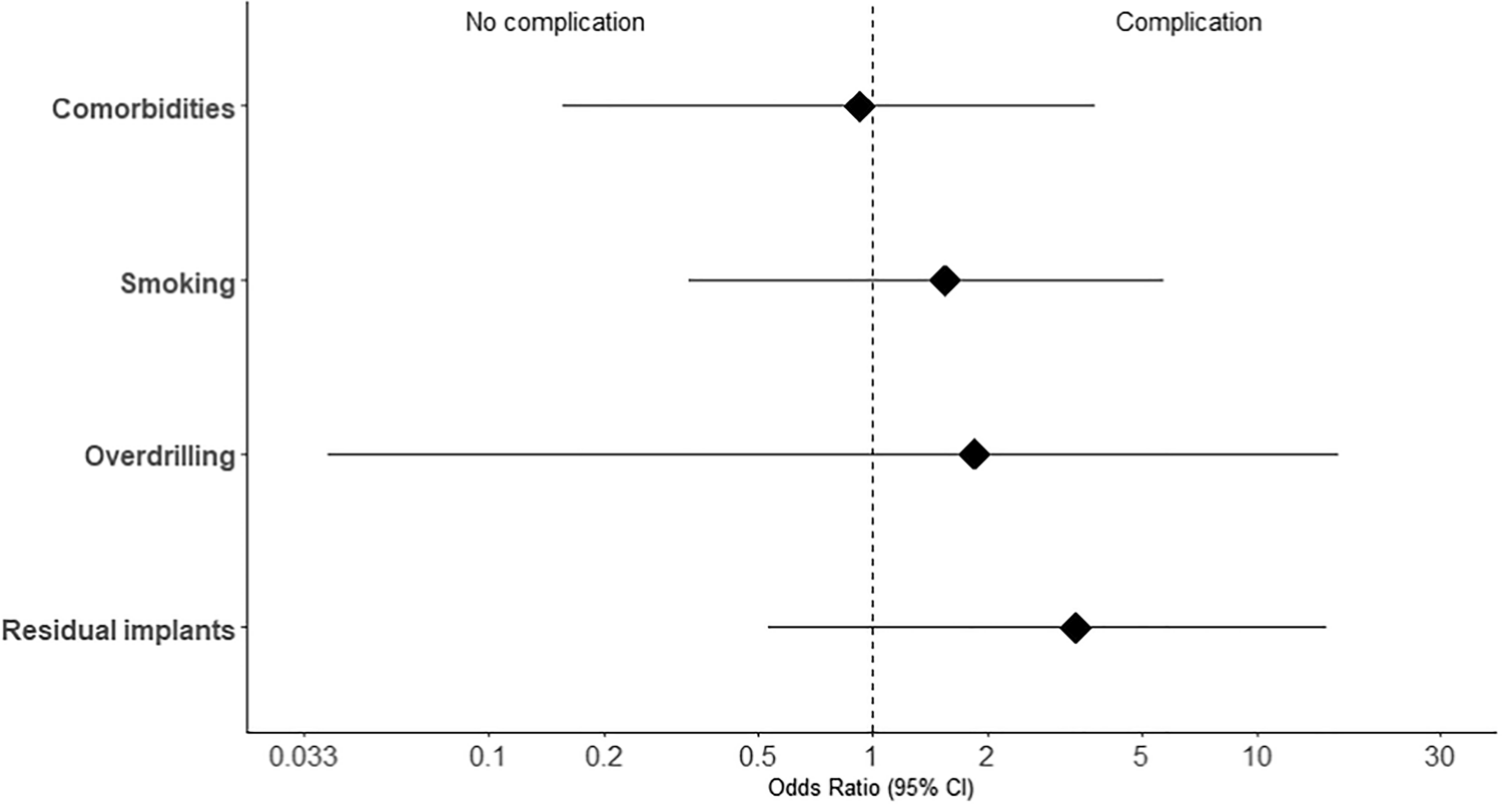
Likelihood of complications represented as odds ratio with 95% confidence intervals

An overview of our secondary outcomes and intraoperative management are shown in Table 2. Overdrilling of damaged implants had to be performed in 4.2% of all procedures. Overall, major complications, including refractures, deep infections, or neurovascular injuries were detected in 2.1% of the procedures. Refractures after IR were detected only in the clavicle (*n* = 3, 3.7%) and olecranon (*n* = 1, 2.5%). Persistent, triggerable pain requiring painkillers following IR was detected in 9% at the six weeks follow-up. Persistent pain was primarily detected after removal of plate osteosynthesis at the olecranon and at the clavicle. At both locations, pain was reported during the final stages of mobilization. At the radius, persistent pain was only described in one instance when a combined IR of the distal radius and ulna took place. Minor complications including wound-healing disorders or superficial bleeding were seen in 5.3% of the cases. Odds ratios were calculated between complications and underlying factors. However, we were not able to detect any statistically significant relationship as demonstrated in Table 3 and Fig. 5.

## Discussion

While elective IR of consolidated fractures is frequently performed in orthopedic and trauma surgery, there is still controversial discussion over its risks and benefits and guidelines or evidence-based recommendations are lacking. Relevant and reliable implant- related functional impairment may result in significant limitations in quality of life since good function of the upper extremity is critical to perform activities of daily life. Therefore, careful discussion of indication for elective hardware is warranted in these patients. In contrast, in patients presenting with unspecific symptoms of discomfort, there is controversy to justify indication for surgical IR. However, as presented in the literature, unspecific symptoms of discomfort represent common and not well- reflected indications for IR. In our study, we investigated the outcome following IR based on three different indications.

The three key results are as follows:

- The primary indication for elective IR in the upper extremity was unspecific symptoms of discomfort
- Unspecific symptoms of discomfort are significantly less likely to be alleviated following IR
- Complications are rare in elective IR

Indications for IR are various and depend on the anatomical region. In this study, the main reasons for IR were unspecific symptoms of discomfort experienced by the patient. We also identified pain and a limitation in the ROM as important indications for IR, which also occurred in combination in some cases.

Our results are in accordance with several recent publications: Hulsmans et al. described irritation as the main indication of IR [6]. Wurm et al. reported limited ROM, pain, and irritation as the main reasons for IR of the upper extremity [7, 8]. Irritation as well as subjective symptoms of discomfort are challenging to verify and quantify, and they render assessment of standardized outcomes difficult. In addition, the results of our study suggest that IR based on unspecific symptoms of discomfort may be associated with frustrating persistence of postoperative symptoms and may cause patient dissatisfaction. Similar to our study, Snoddy et al. also reported on pain (30%) as one of the main objective factors for IR after surgically treated distal radius fractures [9, 10] Additionally, Yuan et al. described cold weather pain, prominence, and general discomfort as additional reasons for IR. Many previous publications have included small patient cohorts with IR in various anatomical regions leading to even smaller sample sizes for the individual anatomic regions [11].

Our second major finding was that patients` satisfaction is dependent on the indication for IR. In this study, only 48.7% of the patients reported a subjective benefit following IR in the upper extremity. Patients were significantly less likely to benefit when the indication was based on unspecific symptoms of discomfort. Therefore, the initial hypothesis that the outcomes are independent of the indication must be rejected. Indeed, patient satisfaction was significantly higher when the indication for IR was based on clinically objective limitations, including symptoms of pain or limitations in ROM. The results of this study suggest that IR of asymptomatic hardware based purely on patients´ subjective requests should be considered carefully and cannot be generally recommended. To our knowledge, this is the first study to investigate this specific relationship.

Williams et al. assessed functional outcomes after IR in a study cohort of 85 patients and detected an improvement in function and ROM in 73% of the upper extremity, which is similar but higher than in our study [12]. Another study that investigated the outcome after IR of superior clavicle plates demonstrated a decrease in pain and an increase in mobilization in their case series [7]. Similarly, in our cohort, the most beneficial outcome for parameters of pain and ROM following IR were observed at the clavicle. Assessment of the ROM in the subgroups before and after IR demonstrated a significant increase in ROM of the wrist after IR of the radius (40%).

Several studies have reported the disadvantages of elective IR. Kempton et al. observed development of pain in 37.5% of patients after removal of asymptomatic hardware [13] In our series, increasing complaints after asymptomatic hardware removal were not observed. Wang et al. recommended to perform elective IR of healed fractures only based upon a patient’s explicit request [14].

Regarding our secondary outcomes, we were able to demonstrate that the rates of postoperative or intraoperative complications were low in our cohort. In the literature, complication rates ranging from 12 to 41% in elective IR have been described. Operative procedures to perform IR are associated with prolonged duration and increased blood loss compared to the initial procedure [15]. Superficial wound infections, iatrogenic injuries, or refractures were rarely detected. Minkowitz et al. already described elective IR in 2007 as a safe procedure with low risks of complications [16, 17]. Williams et al. described in their cohort of 119 patients a complication rate in 10%. Mostly, superficial infections were described [12]. In our study, three refractures (3.7%) were detected following IR at the clavicle and occurred within the first two weeks following IR. The time of IR at the clavicle was approximately two years after open reduction and internal fixation. In our study, 12% of the patients retained partial implant material due to intraoperative decision-making. Similar findings were previously described [10].

Overall, the results of our study suggest that elective IR can be considered a relatively safe procedure. By including only patients from our outpatient setting, we indirectly excluded multimorbid patients with high-risk factors for postoperative complications, for whom IR is performed in an inpatient setting at our hospital, by default. Therefore, potential complications of multimorbid patients were not included in our database.

Finally, there is a financial aspect that can be important for hospitals and healthcare providers [18]. There is limited literature that directly compares the costs of elective IR with other orthopedic procedures. One study claimed that the mean elective IR costs were approximately $5.707 including surgical procedure and anesthesia [19]. Another study reported that costs of operating rooms without surgeons and anesthesia range from $22-$133 per minute [20]. Operative procedures based on unspecific indications may not be reimbursed by health care providers. Such indications may include unspecific symptoms of discomfort or pure psychological unease of the patient to carry a surgical implant viewed as a foreign body. Therefore, meticulous documentation and careful decision-making are important factors in offering elective IR procedures.

### Strengths

To our knowledge, this is the largest cohort of elective IR of the upper extremity in the literature today. Furthermore, our study population was limited to the three predominately operated body regions of the upper extremity, which yielded a large homogeneity among the subjects. A physician of our department performed a consultation of all patients prior to and after surgery including an office visit on the first postoperative day, and six weeks postoperatively. Therefore, detailed documentation and patients’ information was accessible, which resulted in precise assessment of all investigated parameters. In addition, the focus on unspecific symptoms of discomfort was discussed in detail with all patients, which has so far been critically underrepresented in the literature.

### Limitations

This is a retrospective cohort study. We only included patients in this study with a completed follow-up at least six weeks after surgery. Therefore, patients with beneficial resolution of preoperative symptoms and satisfying outcomes after IR may not have returned for a scheduled follow-up visit, which may result in underrepresentation of patients with benefits after IR and may represent a selection bias. In addition, we did not compare long-term outcomes of patients who underwent elective IR to a control group without IR. Complaints occurring more than 6 weeks after surgery were also not detected. Another limitation is the subjective nature of patients` satisfaction. We only concentrated on the patient`s satisfaction or dissatisfaction, as well as the clinical examination postoperatively, as there are no validated objective scores to measure this outcome parameter.

## Conclusion

The results of this study suggest that IR based on unspecific symptoms of discomfort may be associated with frustrating persistence of symptoms, which could cause patient dissatisfaction, and therefore, should not be recommended for every patient. Preoperatively, the patient should be educated about possible postoperative complications following removal of asymptomatic implants and careful decision-making and patient education is recommended. The costs for removal of asymptomatic implants may not be covered and preoperative consent with the insurance companies should be obtained.

However, in patients with symptomatic hardware, including palpable, superficial implants with local tenderness or impaired function, a relief of symptoms, and an improvement in ROM can be expected. The complication rate of IR at the upper extremity was low and thus, it can be considered a relatively safe procedure.

## Abbreviations

CI: Confidence interval
COM: Complications
IR: Implant removal
OR: Odds ratio
SD: Standard deviation
SI: Subjective improvement
ROH: Removal of hardware
ROM: Range of motion
